# Interventional effects of mesenchymal stem cells on epithelial–mesenchymal transition in head and neck squamous cell carcinoma and underlying mechanisms: a systematic review and meta-analysis of *in vitro* studies

**DOI:** 10.3389/fimmu.2025.1705852

**Published:** 2026-01-07

**Authors:** Youhu Wang, Yanli Liu, Wanxian Du, Jing Xue, Jianhua Ruan, Juan Yu, Bin Ma, Xun Li

**Affiliations:** 1 Department of Otorhinolaryngology Head and Neck Surgery, The First Hospital of Lanzhou University, Lanzhou, China; 2 The First School of Clinical Medicine, Lanzhou University, Lanzhou, China; 3 Evidence-Based Medicine Center, School of Basic Medical Sciences, Lanzhou University, Lanzhou, China; 4 Research Center for Medical Device Regulatory Science, Lanzhou University, Lanzhou, China; 5 Industry Center for Evidence-Based Research and Evaluation Standards in Medical Devices of Gansu Province, Lanzhou, China; 6 Department of General Surgery, The First Hospital of Lanzhou University, Lanzhou, China; 7 Key Laboratory Biotherapy and Regenerative Medicine of Gansu Province, Lanzhou, China

**Keywords:** epithelial-mesenchymal transition, head and neck squamous cell carcinoma, mesenchymal stem cells, meta-analysis, tumor microenvironment

## Abstract

**Background:**

MSCs are an important component of the TME and play a key role in tumor progression. Based on existing *in vitro* studies, this research aims to investigate the role of mesenchymal stem cells in the EMT of HNSCC and its related mechanisms.

**Methods:**

According to the PRISMA guidelines, we systematically searched PubMed, Embase, and Web of Science databases for relevant *in vitro* studies up to May 6, 2024. Two trained researchers independently performed literature screening, data extraction, and quality assessment, with cross-checking of results. Any disagreements were resolved through discussion or by consulting a third party. Meta-analysis was conducted using Stata 17 software.

**Results:**

A total of 8 *in vitro* studies were included, involving OSCC, NPC, and TSCC. The meta-analysis results indicate that MSC intervention may be associated with a reduction in the expression of epithelial markers and an increase in mesenchymal markers and related transcription factors in cancer cells, implying a potential role for MSCs in promoting EMT *in vitro*. Furthermore, a preliminary review of the underlying molecular mechanisms suggests that this process may involve the potential regulation of multiple signaling pathways, including NF-κB, PI3K/Akt/mTOR, IL-6R/JAK/STAT3, CXCL8/CXCR2, TGF-β/Smad, and FGF19-FGFR4.

**Conclusions:**

The existing *in vitro* evidence suggests that mesenchymal stem cells may exhibit a potential to promote EMT in HNSCC, potentially regulating tumor progression through multiple signaling pathway networks and providing new potential targets for future therapies targeting the TME. However, more high-quality, standardized *in vivo* and *in vitro* studies are needed to further validate the related mechanisms and therapeutic potential.

## 1 Introduction

Head and neck cancer (HNC) is a group of heterogeneous malignant tumors arising from the mucosal linings of the oral cavity, pharynx, larynx, nose, salivary glands, and esophagus (1, 2). Among these, head and neck squamous cell carcinoma (HNSCC) is the most common pathological type of HNC, accounting for over 90% of HNC cases which originate as squamous cell carcinomas of the oral mucosa, oropharynx, and larynx (3). According to the latest global cancer statistics, HNSCC has become the eighth most common cancer worldwide, with a continuously increasing incidence, making it a major health challenge in urgent need of solutions (4).

Although there have been some advances in HNSCC treatment in recent years, with improvements in standard therapies including surgery and chemoradiotherapy, the overall efficacy of HNSCC treatment remains limited due to high rates of local tumor recurrence and distant metastasis, as well as issues of resistance to chemoradiotherapy. Globally, improvements in overall survival have been disappointing, with five-year survival rates not exceeding 50% (5–8). In light of this predicament, there is an urgent need to explore more effective therapeutic strategies.

In recent years, the bidirectional interactions between tumor cells and the tumor microenvironment (TME) have provided new avenues for HNSCC treatment. As a critical support system for tumor growth, invasion, and metastasis, the TME comprises various non-cancerous components such as fibroblasts, immune cells, and vascular endothelial cells (9). Currently, therapeutic strategies targeting the non-cancerous cells within the TME have become a focal point in HNSCC treatment (10). Among these cells, mesenchymal stem cells (MSCs), owing to their wide availability, multipotent differentiation potential, and immunomodulatory capacity, play an important regulatory role in tumor development and progression (11) and have attracted increasing attention. Studies have shown that MSCs can home to tumor tissues under the guidance of various chemokines and regulate tumor progression through multiple mechanisms including immunomodulation and autocrine/paracrine signaling pathways (12). In particular, MSCs may promote the invasiveness and metastatic ability of tumor cells by inducing epithelial–mesenchymal transition (EMT) (13).

EMT is a biological process by which epithelial cells lose polarity and cell–cell adhesion, transforming into a mesenchymal-like phenotype with migratory and invasive properties; it is a key mechanism driving invasion and metastasis in tumor progression (14, 15). In HNSCC, EMT has been shown to be closely associated with tumor invasiveness, recurrence, and metastasis (16). Meanwhile, *in vitro* studies have demonstrated that MSC-conditioned medium can downregulate epithelial markers (such as E-cadherin and β-catenin) in nasopharyngeal carcinoma cells while upregulating mesenchymal markers and related transcription factors, thereby inducing EMT (17). However, the specific mechanisms by which MSCs regulate EMT in the HNSCC microenvironment and subsequently influence the invasive and migratory properties of cancer cells, remain not fully understood. In particular, how chemokines secreted by tumor cells recruit MSCs to the TME, and how MSCs act on tumor cells and their signaling pathways after homing, thereby driving changes in EMT-related gene expression and phenotype, all require further exploration. Additionally, it has yet to be systematically elucidated whether MSCs from different sources or in different states have differential effects on EMT in HNSCC.

Therefore, this systematic review aims to compile and analyze current *in vitro* studies on the effects of MSCs in HNSCC, to explore whether MSCs can promote cancer cell invasion and migration by inducing EMT, and to identify possible key molecules or signaling pathways involved. By integrating existing evidence, we hope to provide new insights into the metastatic mechanisms of HNSCC and lay the foundation for developing therapies targeting MSCs in the TME.

## 2 Methods

This study strictly followed the PRISMA (Preferred Reporting Items for Systematic Reviews and Meta-Analyses) guidelines (18).

### 2.1 Literature search strategy

We conducted a systematic search of PubMed, Embase, and Web of Science databases from their inception until May 6, 2024. The search strategy used terms related to “head and neck squamous cell carcinoma,” and “mesenchymal stem cells,” as the main keywords, combining MeSH terms with free-text words. Database-specific filters were applied to preliminarily exclude publication types such as letters, reviews, and conference abstracts. To ensure comprehensiveness, we also manually screened the reference lists of included studies. No language or publication date restrictions were applied. The detailed search strategy is provided in **Supplementary Table S1**.

### 2.2 Inclusion and exclusion criteria

We included original studies that met all of the following criteria: (i) *in vitro* studies of HNSCC (including nasopharyngeal, oropharyngeal, hypopharyngeal, laryngeal, oral, salivary gland, nasal, or sinus squamous cell carcinomas); (ii) studies evaluating the effect of MSCs on EMT-related markers, including interventions using either MSCs or MSC-derived products (e.g., conditioned medium, exosomes), with no restrictions on MSC source or status; (iii) no restriction on the type of control; (iv) outcomes included at least one EMT-related marker, such as expression levels of epithelial markers (e.g., E-cadherin) and mesenchymal markers (e.g., β-catenin, N-cadherin, Vimentin, Fibronectin) and/or related transcription factors (e.g., Snail, ZEB family, bHLH family); and (v) full-text available in English or Chinese. Studies were excluded if outcome means and standard deviations could not be obtained (and were not available from authors upon request), or if they were duplicate publications or the full text was unavailable.

### 2.3 Study selection

We imported the search results into EndNote 21 and removed duplicates. Two researchers then independently screened the titles and abstracts according to the inclusion criteria. We first conducted a pilot screening in which each of the two researchers independently screened 10% of the records (randomly selected). Percentage agreement between the two researchers exceeded 75% before proceeding to formal screening. During screening, studies were classified into three groups: include, exclude, and uncertain. Subsequently, the two researchers independently reviewed the full texts of studies that were potentially eligible or uncertain to determine final inclusion. Any disagreements were resolved by consensus or by a third researcher if necessary.

### 2.4 Data extraction

Two researchers independently extracted data from the eligible *in vitro* studies using a pre-designed data extraction form, including: (i) basic information (authors, publication year, HNSCC subtype, cell line); (ii) intervention details (MSC source — e.g., bone marrow, adipose, umbilical cord — and form of intervention: direct/indirect co-culture, conditioned medium, exosomes); (iii) experimental outcomes (changes in EMT markers and related transcription factors, detection methods, relevant signaling pathways). If outcomes were reported at multiple time points, a single reasonable time point was selected after consulting experts. The two researchers cross-checked all extracted data before analysis. Any discrepancies were resolved by consensus or through a third-party consultation.

### 2.5 Quality assessment

Because no standardized tool exists for *in vitro* studies, we referred to a previous study (19) and used the criteria proposed by Golbach et al. for assessing risk of bias in *in vitro* research (20). We evaluated the following 11 factors: cell source, intervention duration, intervention dosage, cell culture conditions, cell viability assessment, experimental procedure, outcome measurement process, randomization and blinding, controls, and standardization of reagents and instruments. Each criterion was rated as “high quality,” “low quality,” or “unclear.” Two researchers independently appraised the quality of the included studies and cross-checked the results. Any disagreements were resolved by consensus or by consulting a third researcher.

### 2.6 Statistical analysis

Meta-analysis was conducted using Stata 17. Fold-change data were log-transformed before analysis. Subgroup analyses were performed according to biomarker type and detection method. Because measurement scales were consistent within detection-method subgroups, the mean difference (MD) was used as the effect size for continuous variables, and 95% confidence intervals (CI) were calculated. Between-study heterogeneity was assessed using the Cochrane Q test and the I² statistic. Considering the inherent heterogeneity in cell lines and experimental conditions, a random-effects model was applied for all pooled analyses. Sensitivity analysis was conducted using a leave-one-out approach. A P value < 0.05 was considered statistically significant.

## 3 Result

### 3.1 Literature search and selection results

**Figure 1:**
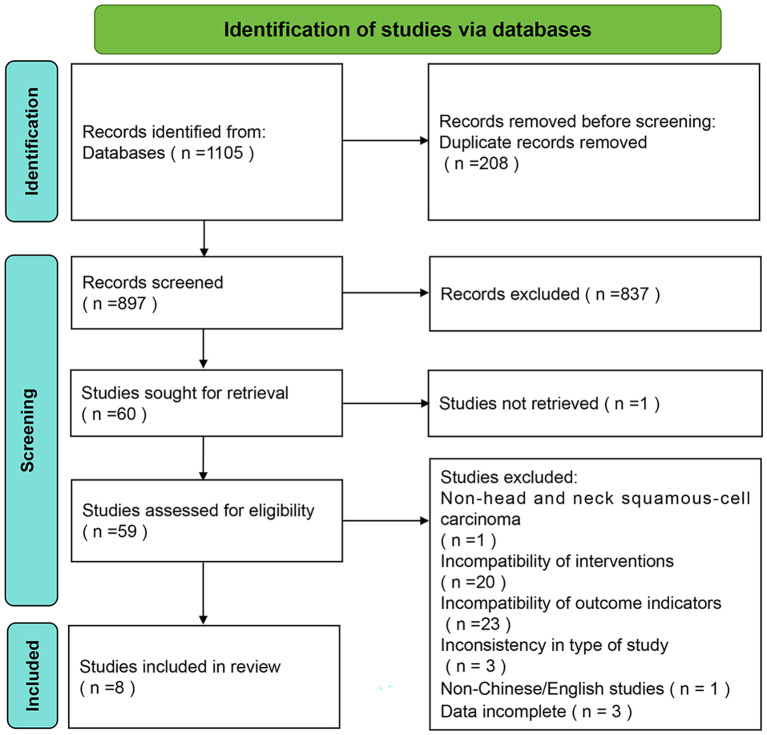
PRISMA flow diagram of study screening and selection.

Using the search strategy described in the Methods, a total of 1105 relevant publications were retrieved, and 897 studies were included in the screening after removing duplicates. Detailed screening was performed according to the nerf criteria, and after reading the full text of 59 potentially relevant papers, 8 cellular experiments were finally included (17, 21–27). **Figure 1** illustrates the screening process of the literature.

### 3.2 Basic characteristics of included studies

A total of 8 studies were included in this review (17, 21–27). These studies covered three subtypes of HNSCC: 5 studies (21, 23–26) focused on oral squamous cell carcinoma (OSCC), 2 studies (17, 22) on nasopharyngeal carcinoma (NPC), and 1 study (27) on tongue squamous cell carcinoma (TSCC). All studies used MSCs as the intervention. In 6 studies (17, 22–24, 26, 27), the MSCs were derived from human bone marrow; in 1 study (21), the MSCs were derived from human OSCC tumor tissue; and 1 study (25) did not clearly report the MSC source. Regarding the culture methods between MSCs and HNSCC cells, 5 studies (22–24, 26, 27) used MSC-conditioned medium, 1 study (21) used a combination of indirect co-culture and conditioned medium, 1 study (25) used direct co-culture, and 1 study (17) used MSC-derived exosomes.

Our analysis focused on changes in EMT-related marker expression as a result of MSC exposure. For epithelial markers, all included studies used E-cadherin as the epithelial marker; in addition, one study (22) also examined β-catenin. For mesenchymal markers, one study (25) assessed only Vimentin, whereas the others assessed both Vimentin and N-cadherin. The transcription factors evaluated included Snail, Twist, MMP-2, MMP-9, ZEB1, and ZEB2. Most experiments detected marker changes via Western blotting (17, 21) or quantitative PCR (22–24, 26, 27), and one study (25) used immunohistochemistry for some markers. The detailed characteristics of all included studies are presented in **Supplementary Table S2**.

### 3.3 Risk of bias

**Figure 2:**
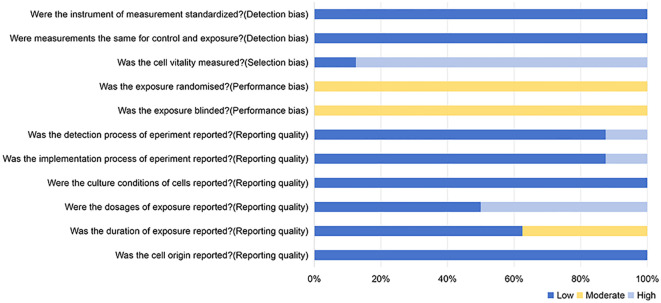
Risk of bias assessment.

The results of the risk of bias assessment are shown in **Figure 2**. Among the included studies, 87.5% (7/8) did not perform a cell viability assay, 50% (4/8) did not report the intervention dosage, and 37.5% (3/8) did not clearly report the intervention duration. Additionally, 12.5% (1/8) did not report the outcome measurement process, and 12.5% (1/8) did not report the experimental implementation process.

### 3.4 Meta-analysis results

#### 3.4.1 Effect of MSCs on EMT in OSCC

**Figure 3:**
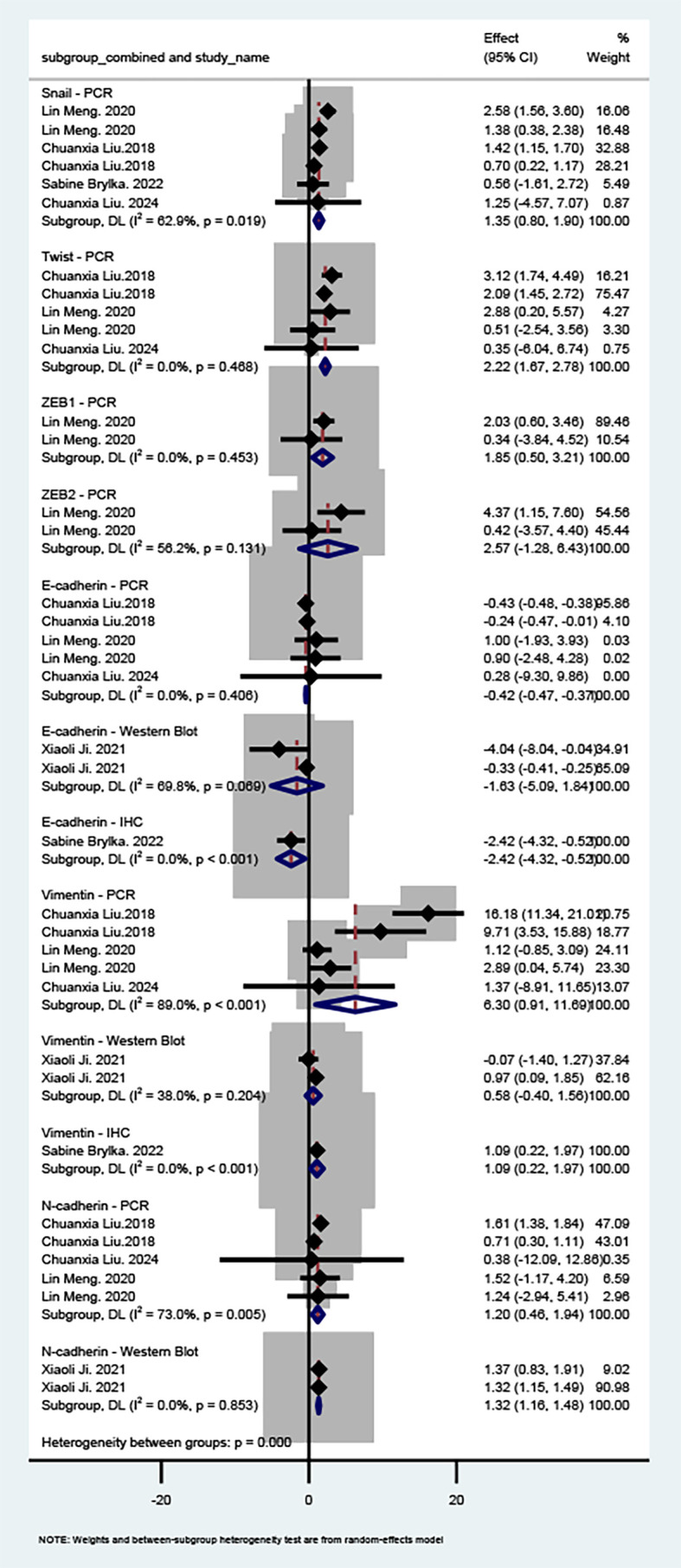
Forest plot of the effect of MSCs on EMT markers in OSCC. Studies are stratified by molecular marker and detection method. Each line represents one study plotted according to the Weighted Mean Difference (WMD). The squares show the WMD for each study; the diamond represents the pooled WMD. Weights are from the random-effects model. WMD: Weighted Mean Difference.

Of the 8 included studies, 5 (21, 23–26) focused on OSCC. **Figure 3** presents the meta-analysis results for the OSCC subgroup. For epithelial markers, both PCR and IHC analyses showed reduced E-cadherin expression (PCR MD = −0.42, 95% CI: −0.47 to −0.37; IHC MD = -2.42, 95% CI: -4.32 to -0.52). Although the Western blot subgroup did not reach statistical significance, the effect direction indicated a downward trend. For mesenchymal markers, Vimentin expression was increased in both PCR (MD = 6.30, 95% CI: 0.91 to 11.69) and IHC (MD = 1.09, 95% CI: 0.22 to 1.97) analyses, while the Western blot subgroup showed an upward but non-significant trend. N-cadherin was consistently elevated in both the PCR and Western blot subgroups (PCR MD = 1.20, 95% CI: 0.46 to 1.94; Western blot MD = 1.32, 95% CI: 1.16 to 1.48).

At the transcriptional level, Snail mRNA was significantly upregulated (MD = 1.35, 95% CI: 0.80 to 1.90). Although Twist and the ZEB family showed upward effect directions, neither reached statistical significance. Sensitivity analysis (**Supplementary Figure S1**) indicated that sequential exclusion of studies with high heterogeneity or large weights caused some fluctuation in pooled effect sizes; however, the overall effect direction remained consistent, and confidence intervals did not cross the null line, suggesting reasonable robustness. Taken together, although some protein-level findings and downstream transcription factors did not reach statistical significance, key initiators such as Snail and core markers (e.g., increased Vimentin, decreased E-cadherin by IHC) demonstrated consistent biological trends. Current *in vitro* evidence suggests that MSCs may exert a pro-EMT effect in OSCC, though further high-quality studies are needed to validate these observations.

#### 3.4.2 Effect of MSCs on EMT in NPC

**Figure 4:**
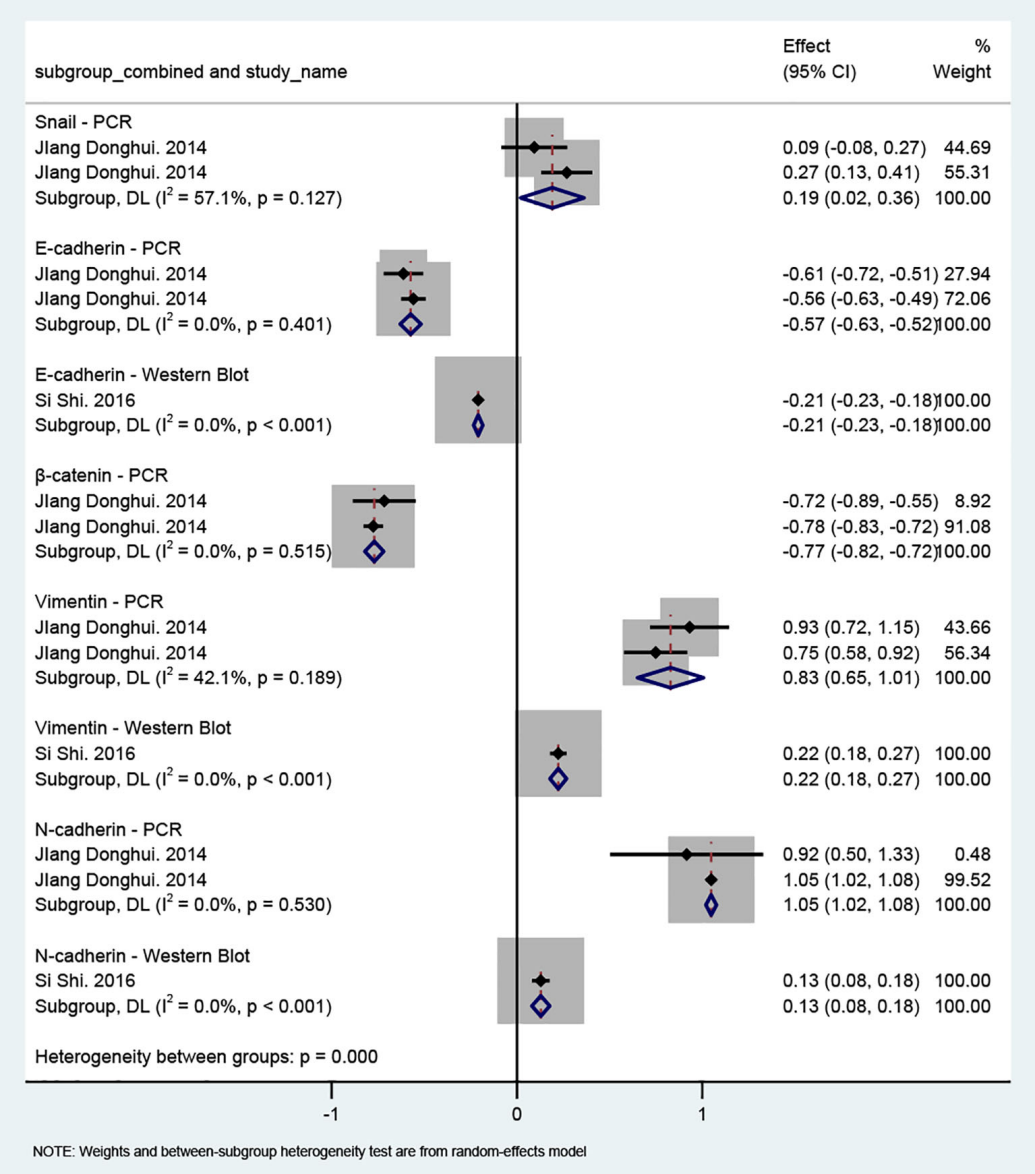
Forest plot of the effect of MSCs on EMT markers in NPC. Studies are stratified by molecular marker and detection method. Each line represents one study plotted according to the Weighted Mean Difference (WMD). The squares show the WMD for each study; the diamond represents the pooled WMD. Weights are from the random-effects model. WMD: Weighted Mean Difference.

Two of the included studies (17, 22) focused on NPC. **Figure 4** illustrates the effects of MSCs on EMT-related markers and transcription factors in NPC. For epithelial markers, both PCR (MD = −0.57, 95% CI: −0.63 to −0.52) and Western blot analyses (MD = −0.21, 95% CI: −0.23 to −0.18) demonstrated decreased E-cadherin expression. β-catenin mRNA levels also showed a corresponding decline (MD = −0.77, 95% CI: −0.82 to −0.72). In contrast, mesenchymal markers exhibited clear upregulation across different measurement methods: Vimentin expression increased in both PCR (MD = 0.83, 95% CI: 0.65 to 1.01) and Western blot analyses (MD = 0.22, 95% CI: 0.18 to 0.27); N-cadherin showed concordant elevation at both the mRNA level (MD = 1.05, 95% CI: 1.02 to 1.08) and protein level (MD = 0.13, 95% CI: 0.08 to 0.18). In addition, Snail expression was modestly increased (MD = 0.19, 95% CI: 0.02 to 0.36). Sensitivity analyses (**Supplementary Figure S2**) indicated that the effect directions remained stable after sequential exclusion of individual studies, and none of the confidence intervals crossed the null value, suggesting reasonable robustness. Overall, MSCs appear to promote EMT in the NPC microenvironment. However, given the limited number of available studies, further high-quality research is needed to confirm the generalizability of this conclusion.

#### 3.4.3 Effect of MSCs on EMT in TSCC

**Figure 5:**
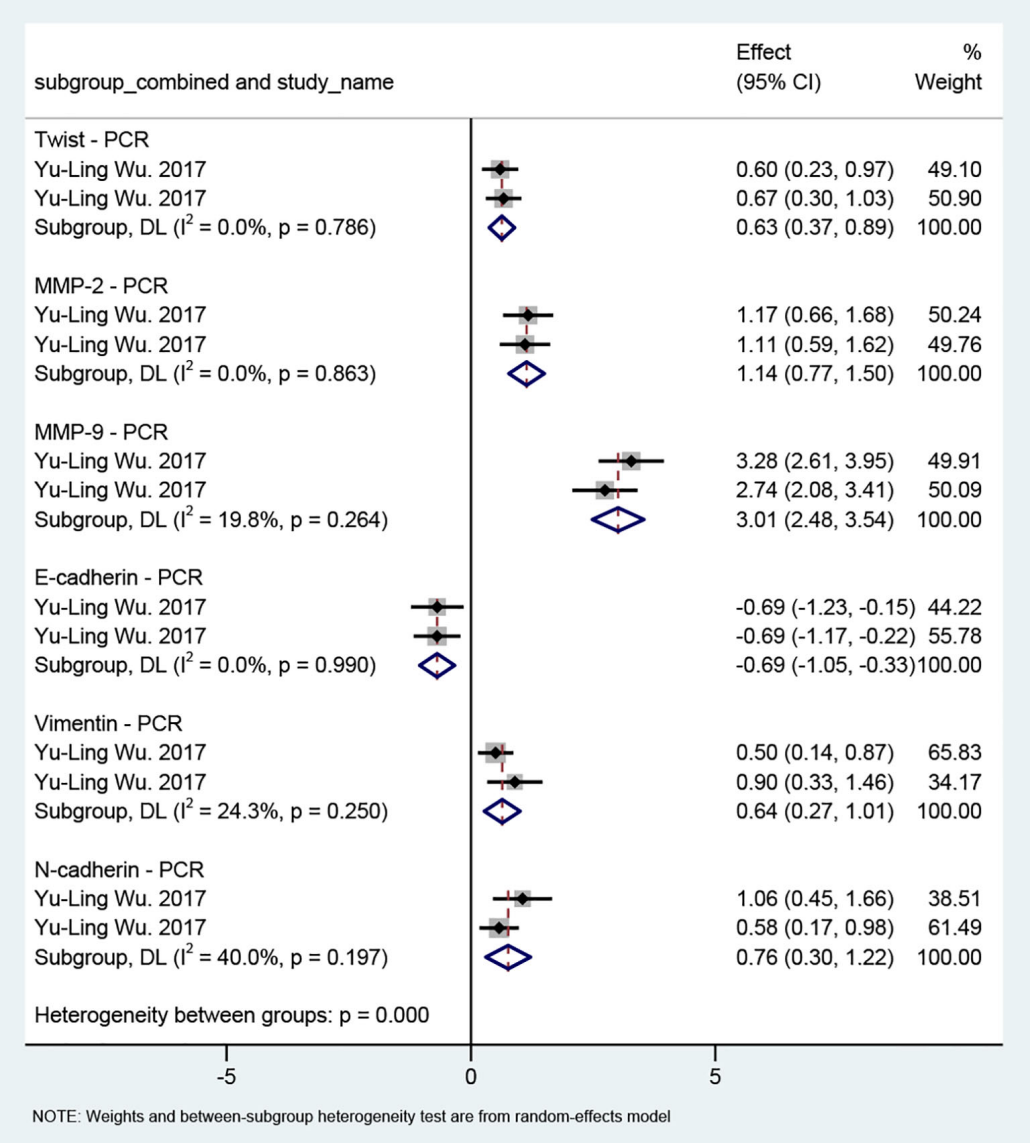
Forest plot of the effect of MSCs on EMT markers in TSCC. Studies are stratified by molecular marker and detection method. Each line represents one study plotted according to the Weighted Mean Difference (WMD). The squares show the WMD for each study; the diamond represents the pooled WMD. Weights are from the random-effects model. WMD: Weighted Mean Difference.

Only one study (27) evaluated the effect of MSCs on EMT in TSCC; therefore, the results are presented based on single-study effect sizes (**Figure 5**), and no sensitivity analysis was performed. PCR data showed a downward trend in the epithelial marker E-cadherin (MD = −0.69, 95% CI: −1.05 to −0.33), while the mesenchymal markers Vimentin (MD = 0.64, 95% CI: 0.27 to 1.01) and N-cadherin (MD = 0.76, 95% CI: 0.30 to 1.22) both exhibited upward trends. Regarding key EMT transcription factors, Twist expression was increased (MD = 0.63, 95% CI: 0.37 to 0.89). In addition, MMP proteins, which are closely associated with matrix degradation and cellular invasion, were markedly elevated. Both MMP-2 (MD = 1.14, 95% CI: 0.77 to 1.50) and MMP-9 (MD = 3.01, 95% CI: 2.48 to 3.54) showed significant upregulation. Overall, these findings suggest that MSCs may also promote EMT in TSCC. However, as the evidence is derived from a single study, further research is needed to verify this potential effect.

Synthesizing the findings from OSCC, NPC, and TSCC, the evidence suggests that MSCs may exert a pro-EMT effect within the microenvironment of head and neck squamous cell carcinoma (HNSCC), despite the heterogeneity across tumor subtypes and measurement methods. Across all analyzed subgroups, the most prominent phenotypic pattern consisted of downregulation of epithelial markers accompanied by upregulation of mesenchymal markers and invasion-related proteins. In addition, the consistently elevated expression of key transcription factors across subgroups further indicates that MSCs may initiate the EMT program by activating upstream transcriptional networks. However, these overall findings are derived from *in vitro* studies, and the number of available studies for each cancer type is limited (most subgroups included only 1–2 studies), constraining statistical precision and external generalizability. Therefore, the conclusions of this study should be interpreted as directional evidence across cancer types, indicating that MSCs may induce EMT-related phenotypic changes in HNSCC, rather than as precise quantitative estimates of effect size. Further high-quality and methodologically standardized studies are needed to validate this emerging trend.

### 3.5 Roles and mechanisms of MSCs in EMT of HNSCC

**Figure 6:**
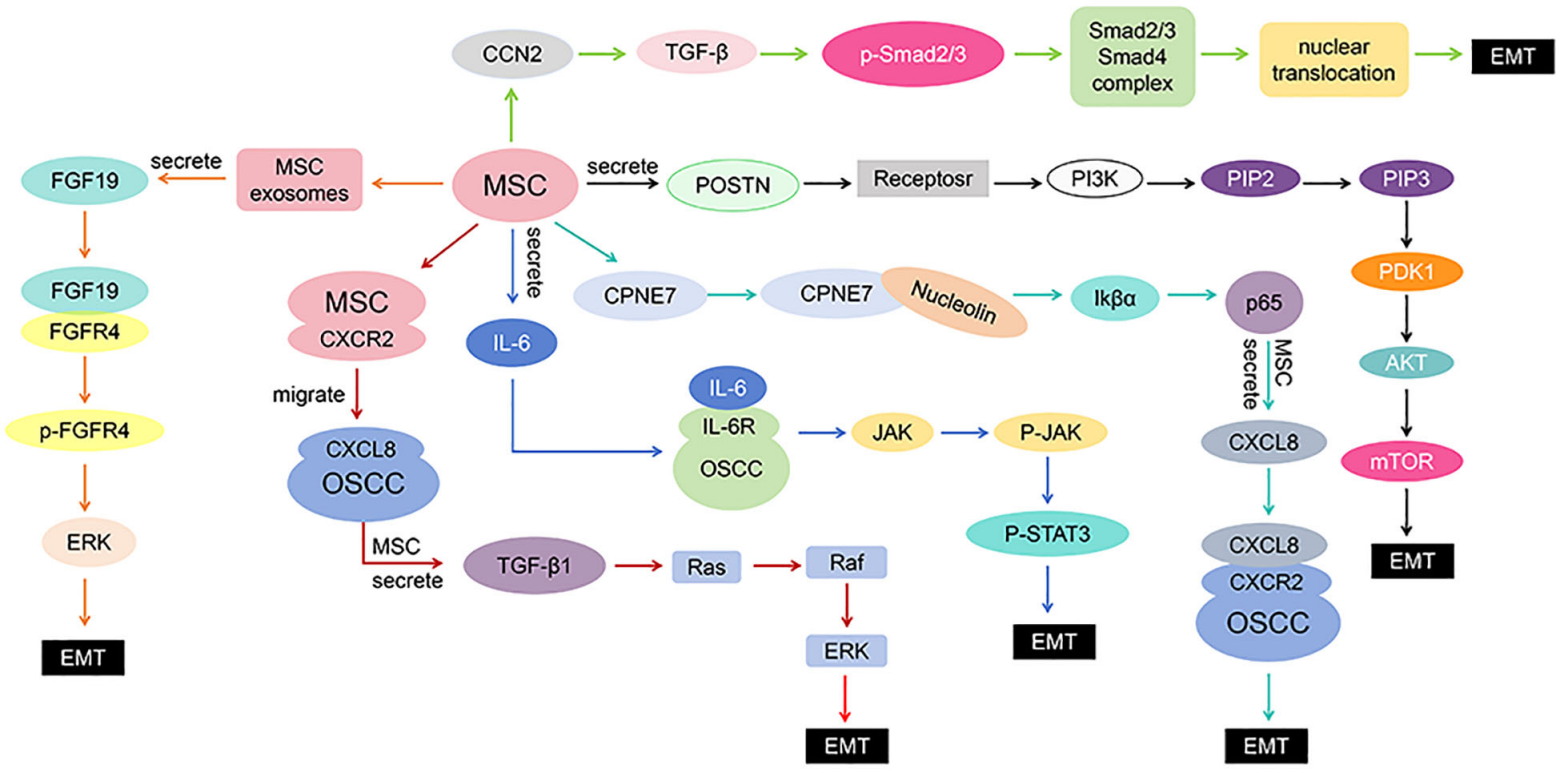
Signaling pathways diagram in the included studies.

**Table 1** summarizes the six distinct signaling pathways identified in the included studies, and a simplified schematic diagram (**Figure 6**) is provided to visually illustrate their mechanisms of action.

**Table 1:** Signaling pathways in the included studies.

First author, year	Head and neck squamous cell carcinoma, HNSCC	Relevant signaling pathways/mechanisms
Xiaoli Ji, 2021 (21)	oral squamous cell carcinoma, OSCC	CPNE7 controls CXCL8 secretion through the NF-κB signaling pathway
JIang Donghui, 2014 (22)	Nasopharyngeal carcinoma, NPC	Not clearly reported
Chuanxia Liu, 2018 (23)	oral squamous cell carcinoma, OSCC	POSTN‐mediated PI3K/Akt/mTOR pathway
Chuanxia Liu, 2024 (24)	oral squamous cell carcinoma, OSCC	IL-6R/STAT3/JAK pathway
Sabine Brylka, 2022 (25)	oral squamous cell carcinoma, OSCC	Not clearly reported
Lin Meng, 2020 (26)	oral squamous cell carcinoma, OSCC	CXCL8-CXCR2 pathway TGF‐β1/Ras/Raf/Erk signaling pathway
Si Shi, 2016 (17)	Nasopharyngeal carcinoma, NPC	FGF19-FGFR4 signaling pathway
Yu-Ling Wu, 2017 (27)	Tongue squamous cell carcinoma, TSCC	MSC-derived CCN2 regulates cancer cells through the TGF-β signaling pathway

#### 3.5.1 Roles and mechanisms of MSCs in EMT of OSCC

In total, 5 OSCC studies were included, and 4 of these examined the roles and mechanisms of MSCs on OSCC cells. Current evidence suggests that MSCs affect EMT in OSCC by secreting multiple bioactive factors and activating various signaling pathways.

Xiaoli Ji et al. (21) showed that MSCs enhance the migratory and invasive capabilities of OSCC cells by upregulating CPNE7, which activates the NF-κB signaling pathway and increases the secretion of CXCL8. Highly expressed CPNE7 in MSCs interacts with nucleolin on the cell surface and then binds IκBα, promoting the phosphorylation of IκBα and p65, leading to the dissociation of the NF-κB complex. Phosphorylated p65 translocates into the nucleus and regulates the secretion of CXCL8. MSC-derived CXCL8 binds to the CXCR2 receptor on OSCC cells, promoting EMT in OSCC cells.

Chuanxia Liu et al. (23) demonstrated that the POSTN-mediated PI3K/Akt/mTOR pathway plays an essential role in the progression of OSCC. POSTN (periostin) is an important extracellular matrix protein secreted by MSCs. By binding to receptors on the surface of OSCC cells, POSTN triggers the activation of PI3K. Activated PI3K catalyzes the conversion of PIP2 into PIP3, which subsequently recruits PDK1 and Akt, leading to Akt phosphorylation and activation. Activated Akt further activates mTOR, thus promoting proliferation, anti-apoptotic responses, and EMT in OSCC cells. In a subsequent study (24), Chuanxia Liu et al. further confirmed the involvement of the IL-6R/JAK/STAT3 pathway. IL-6 secreted by MSCs binds to IL-6R on OSCC cells, activating the JAK/STAT3 signaling pathway. Signal transduction mediated by IL-6R results in JAK phosphorylating STAT3, which subsequently translocates into the nucleus to induce EMT in OSCC cells. Additionally, Lin Meng et al. (26) revealed the importance of the CXCL8-CXCR2 pathway and the TGF-β1/Ras/Raf/ERK signaling pathway in OSCC development. MSC-derived CXCL8 binds to CXCR2 receptors on OSCC cells, promoting intracellular signaling cascades leading to increased secretion of TGF-β1 by MSCs. As a potent pro-metastatic factor, TGF-β1 binds to its receptors, activating the Ras-Raf-ERK signaling pathway. The activated ERK then enters the nucleus, promoting EMT in OSCC cells.

#### 3.5.2 Roles and mechanisms of MSCs in EMT of NPC

Two studies investigated NPC, of which one explored the mechanism. Si Shi et al. (17) found that the FGF19-FGFR4 signaling pathway plays an important role in NPC progression. FGF19 was highly expressed in MSC-derived exosomes, and as a ligand, FGF19 binds to FGFR4 on NPC cells, triggering downstream signaling cascades via phosphorylation and activating the ERK pathway. Activated ERK then translocates to the nucleus and promotes EMT in NPC cells.

#### 3.5.3 Roles and mechanisms of MSCs in EMT of TSCC

For TSCC, one study was conducted. Yu-Ling Wu et al. (27) found that MSC-secreted CCN2 (Connective Tissue Growth Factor 2, also known as CTGF) can regulate the invasion and metastasis of TSCC cells via the TGF-β signaling pathway. MSC-derived CCN2 induces the TGF-β signaling cascade, promoting the phosphorylation of Smad2/3. After the Smad complex enters the nucleus, it induces EMT in TSCC cells and enhances the invasive capacity of the tumor cells.

Together, these findings indicate that MSCs may regulate the progression of HNSCC through multiple signaling networks. Meanwhile, different subtypes of HNSCC may have distinct pathway activation profiles and regulatory mechanisms. These differences may be related to the tissue microenvironment, gene expression patterns, and tumor characteristics. Future studies can further explore the interactions among these pathways and how they collectively influence key processes such as cancer cell migration, invasion, and immune evasion. This will help provide a theoretical basis for precision therapy of HNSCC and guide the development of targeted therapies.

### 3.6 Publication bias

Egger’s test did not indicate any significant publication bias (p = 0.078).

## 4 Discussion

In this study, we conducted a systematic review and meta-analysis to synthesize existing *in vitro* evidence and explore the potential regulatory role of mesenchymal stem cells (MSCs) in epithelial–mesenchymal transition (EMT) in head and neck squamous cell carcinoma (HNSCC). The pooled analysis indicated that, under co-culture with MSCs, HNSCC cells, including OSCC, NPC, and TSCC, generally showed downregulation of epithelial markers (E-cadherin), upregulation of mesenchymal markers (Vimentin, N-cadherin), and increased expression of EMT-related transcription factors. This phenotypic change suggests that tumor cells may lose polarity and acquire greater invasive and metastatic potential (28). It should be noted, however, that these conclusions are based on only eight *in vitro* studies meeting the inclusion criteria. Given the extremely small sample size and methodological heterogeneity among the studies, the current evidence should be regarded as exploratory rather than definitive for clinical translation.

At the mechanistic level, pathways such as NF-κB, TGF-β, STAT3, and FGFR do not operate independently, but instead interact and cascade to collectively drive EMT, ultimately converging on core transcription factor networks including Snail, Slug, Twist, and ZEB, which promote loss of cell adhesion and increased migratory capacity (29). Previous studies have shown that STAT3 can integrate TGF-β and Ras signaling to upregulate Snail (30), while the TNF-α–NF-κB axis stabilizes Slug protein and enhances EMT (31). TGF-β can also induce EMT-related transcription factors through both SMAD-dependent and SMAD-independent pathways (32). Cytokines, metabolic states, and mechanical stress within the tumor microenvironment may act as upstream regulatory nodes, thereby modulating the effects of MSCs on cancer cells. These observations suggest that MSC-induced EMT is more likely driven by an integrated network across multiple pathways rather than a single linear signal (29). In addition, recent studies indicate that mTOR, particularly nuclear mTOR, plays a critical role in EMT-related metabolic and transcriptional regulation (33, 34). The amino acid–mTOR axis can regulate cellular energy status and influence EMT programs (35). Although these studies did not specifically focus on MSC-induced EMT, they highlight mTOR as a potential key hub through which MSCs may regulate EMT.

Evidence across multiple tumor types suggests that MSCs commonly promote EMT in epithelial cancer cells and enhance their invasiveness. In breast cancer, Klopp et al. (36) reported that MSC-conditioned medium downregulated E-cadherin, upregulated N-cadherin, and accelerated tumor formation *in vivo*. In lung cancer, tumor-associated MSCs similarly induced EMT features and promoted metastasis (37). These observations indicate that, whether in breast cancer, lung cancer, or head and neck squamous cell carcinoma as examined in this study, MSCs exhibit consistent pro-EMT and pro-invasive effects (36, 37). This consistency suggests a potential common mechanism in which MSCs, once recruited and “educated” by the tumor to become tumor-associated MSCs, acquire functions that promote malignant progression across different tumor contexts (38). The studies included in the present review also almost uniformly reported pro-EMT effects of MSCs. However, this conclusion contrasts with reports of the dual role of MSCs as anti-tumor agents, in which genetically engineered or otherwise modified MSCs can inhibit tumor growth (39–43). Notably, Böhrnsen et al. (44) observed in an HNSCC model that co-culture reduced E-cadherin expression but simultaneously downregulated other EMT markers, including Wnt3, MMP14, and β-catenin, leading them to conclude that MSCs may inhibit EMT. This study was not included in the present analysis due to data extraction limitations. The discrepancies between pro- and anti-EMT outcomes may relate to experimental conditions, the properties of the cell lines, or the selection of markers, highlighting the need for more standardized, high-quality studies to further investigate and validate the effects of MSCs on EMT in HNSCC.

Our analysis revealed widespread changes in EMT markers, but this does not imply an “all-or-none” transition. EMT is a dynamic process, and we speculate that MSC-induced EMT might be staged or incomplete; tumor cells may not need to lose all epithelial features to gain enhanced invasiveness. This is supported by findings in lung cancer by Yan et al. (37): even with only transcription factors such as Snail/Slug upregulated and E-cadherin not completely lost, the migratory and metastatic potential of lung cancer cells increased significantly. This suggests that EMT exists on a continuum; MSCs might induce tumor cells into an intermediate “hybrid” state, balancing migration and proliferation, which is more conducive to metastasis formation (45).

This study has several important limitations. First, the number of included original studies was limited, with only eight studies in total, and most cancer-type subgroups contained just one or two studies. This constrains the precision of effect size estimates and reduces the statistical power for heterogeneity analysis and publication bias assessment. Second, *in vitro* studies inherently involve certain variability in experimental conditions, including the source of MSCs (bone marrow or tumor-derived), co-culture methods (direct or indirect contact, exosomes, conditioned medium), treatment doses and duration, as well as inconsistencies in detection methods across studies. Such methodological heterogeneity may influence both the magnitude and comparability of effect sizes, although we attempted to control for these factors through subgroup and sensitivity analyses. In addition, normalization methods for protein expression varied across studies, and some studies lacked systematic correction for cell viability or cell number, which may confound EMT marker changes with overall cell state. Furthermore, the current evidence is primarily derived from two-dimensional *in vitro* co-culture systems, which cannot fully capture the complexity of the tumor microenvironment, including the combined effects of immune cells, stromal components, and mechanical forces. Consequently, the biological and clinical generalizability of these findings remains limited.

## 5 Conclusion

This systematic review of current *in vitro* evidence suggests that MSCs may promote EMT in HNSCC and act through multiple signaling pathways. This observation implies that MSCs within the tumor microenvironment may influence tumor invasion and metastasis, highlighting potential clinical relevance. Although the number of available studies remains limited, our analysis provides an integrated framework for further investigation of MSC–HNSCC interactions. As more high-quality studies accumulate and mechanistic insights deepen, targeting MSC-related pathways may emerge as a novel strategy for anti-metastatic therapy in the comprehensive treatment of HNSCC.

## Data Availability

The original contributions presented in the study are included in the article/**Supplementary Material**. Further inquiries can be directed to the corresponding authors.
