# Correction: Interventional effects of mesenchymal stem cells on epithelial-mesenchymal transition in head and neck squamous cell carcinoma and underlying mechanisms: a systematic review and meta-analysis of *in vitro* studies

**DOI:** 10.3389/fimmu.2026.1786161

**Published:** 2026-01-19

**Authors:** Youhu Wang, Yanli Liu, Wanxian Du, Jing Xue, Jianhua Ruan, Juan Yu, Bin Ma, Xun Li

**Affiliations:** 1 Department of Otorhinolaryngology Head and Neck Surgery, The First Hospital of Lanzhou University, Lanzhou, China; 2 The First School of Clinical Medicine, Lanzhou University, Lanzhou, China; 3 Evidence-Based Medicine Center, School of Basic Medical Sciences, Lanzhou University, Lanzhou, China; 4 Research Center for Medical Device Regulatory Science, Lanzhou University, Lanzhou, China; 5 Industry Center for Evidence-Based Research and Evaluation Standards in Medical Devices of Gansu Province, Lanzhou, China; 6 Department of General Surgery, The First Hospital of Lanzhou University, Lanzhou, China; 7 Key Laboratory Biotherapy and Regenerative Medicine of Gansu Province, Lanzhou, China

**Keywords:** epithelial-mesenchymal transition, head and neck squamous cell carcinoma, mesenchymal stem cells, meta-analysis, tumor microenvironment

The funders Hospital Fund of the First Hospital of Lanzhou University (ldyyyn2021-25), Program of Biomedical Research Center of Northwest Minzu University (GC202101), and Natural Science Foundation of Gansu Province (21JR7RA363) to the authors were erroneously omitted.

The original version of this article has been updated.

