# Supplementary material for: Interventional effects of mesenchymal stem cells on epithelial–mesenchymal transition in head and neck squamous cell carcinoma and underlying mechanisms: a systematic review and meta-analysis of in vitro studies
Source: Front Immunol. 2026 Jan 7;16:1705852. doi: 10.3389/fimmu.2025.1705852 (PMC12862828; doi:10.3389/fimmu.2025.1705852)
Supplement: Supplementary file 1 [file DataSheet1.docx]

Supplementary Material

# Supplementary Tables

## Supplementary Table 1 Search strategy

|  | | PubMed | | |  |
| --- | --- | --- | --- | --- | --- |
| #1 | | "Squamous Cell Carcinoma of Head and Neck"[MeSH Terms] OR "head and neck squamous cell carcinom*"[Title/Abstract] OR "Squamous Cell Carcinoma of the Head and Neck"[Title/Abstract] OR "Squamous Cell Carcinoma of the Larynx"[Title/Abstract] OR "Laryngeal Squamous Cell Carcinom*"[Title/Abstract] OR "Squamous Cell Carcinoma of Larynx"[Title/Abstract] OR "Squamous Cell Carcinoma of the Nasal Cavity"[Title/Abstract] OR "Oral Tongue Squamous Cell Carcinom*"[Title/Abstract] OR "Hypopharyngeal Squamous Cell Carcinom*"[Title/Abstract] OR "Oral Cavity Squamous Cell Carcinom*"[Title/Abstract] OR "Squamous Cell Carcinoma of the Mouth"[Title/Abstract] OR "Oropharyngeal Squamous Cell Carcinom*"[Title/Abstract] OR "HNSCC"[Title/Abstract] | | |  |
| #2 | | "Nasopharyngeal Neoplasms"[MeSH Terms] OR "Nasopharyngeal Neoplas*"[Title/Abstract] OR "nasopharynx neoplas*"[Title/Abstract] OR "Cancer of Nasopharynx"[Title/Abstract] OR "nasopharynx cance*"[Title/Abstract] OR "nasopharyngeal cance*"[Title/Abstract] OR "Cancer of the Nasopharynx"[Title/Abstract] OR "nasopharyngeal carcinom*"[Title/Abstract] OR "nasopharynx tum*"[Title/Abstract] OR "nasopharyngeal tum*"[Title/Abstract] OR "rhinopharyngeal tum*"[Title/Abstract] OR "nasopharynx tum*"[Title/Abstract] OR "neoplasm of the nasopharynx"[Title/Abstract] OR "tumor of the nasopharynx"[Title/Abstract] | | |  |
| #3 | | "Oropharyngeal Neoplasms"[MeSH Terms] OR "oropharyngeal neoplas*"[Title/Abstract] OR "oropharynx neoplas*"[Title/Abstract] OR "oropharnyx cance*"[Title/Abstract] OR "oropharyngeal cance*"[Title/Abstract] OR "Cancer of the Oropharynx"[Title/Abstract] OR "tonsil neoplas*"[Title/Abstract] OR "tonsillar neoplas*"[Title/Abstract] OR "Cancer of Tonsil"[Title/Abstract] OR "Cancer of the Tonsil"[Title/Abstract] OR "tonsil cance*"[Title/Abstract] OR "tonsillar cance*"[Title/Abstract] OR “tonsil carcinom*”[Title/Abstract] OR "oropharyngeal tum*"[Title/Abstract] OR "oropharynx tum*"[Title/Abstract] | | |  |
| #4 | | "Hypopharyngeal Neoplasms"[MeSH Terms] OR "hypopharyngeal neoplas*"[Title/Abstract] OR "hypopharyngeal cance*"[Title/Abstract] OR "hypopharyngeal carcinom*"[Title/Abstract] OR "hypopharynx cance*"[Title/Abstract] OR "hypopharyngeal tum*"[Title/Abstract] OR "hypopharynx tum*"[Title/Abstract] | | |  |
| #5 | | "Laryngeal Neoplasms"[MeSH Terms] OR "laryngeal neoplas*"[Title/Abstract] OR "larynx neoplas*"[Title/Abstract] OR "Cancer of Larynx"[Title/Abstract] OR "larynx cance*"[Title/Abstract] OR "laryngeal cance*"[Title/Abstract] OR "Cancer of the Larynx"[Title/Abstract] OR "throat cance*"[Title/Abstract] OR "laryngeal carcinom*"[Title/Abstract] OR "laryngocarcinom*"[Title/Abstract] OR "glottic tum*"[Title/Abstract] OR "laryngeal tum*"[Title/Abstract] OR "larynx tum*"[Title/Abstract] OR "neoplastic laryn*"[Title/Abstract] OR "subglottic tum*"[Title/Abstract] OR "supraglottic neoplas*"[Title/Abstract] OR "vocal cord tum*"[Title/Abstract] OR "vocal cord neoplas*"[Title/Abstract] | | |  |
| #6） | | "Mouth Neoplasms"[MeSH Terms] OR "mouth neoplas*"[Title/Abstract] OR "oral neoplas*"[Title/Abstract] OR "Cancer of Mouth"[Title/Abstract] OR "oral cance*"[Title/Abstract] OR "Cancer of the Mouth"[Title/Abstract] OR "mouth cance*"[Title/Abstract] OR "gingival neoplas*"[Title/Abstract] OR "congenital epuli*"[Title/Abstract] OR "oral leukoplaki*"[Title/Abstract] OR "oral leukokeratos*"[Title/Abstract] OR "oral keratos*"[Title/Abstract] OR "hairy leukoplaki*"[Title/Abstract] OR "oral hairy leukoplaki*"[Title/Abstract] OR "lip neoplas*"[Title/Abstract] OR "Cancer of Lip"[Title/Abstract] OR "lip cance*"[Title/Abstract] OR "palatal neoplas*"[Title/Abstract] OR "salivary gland neoplas*"[Title/Abstract] OR "salivary gland lymphadenom*"[Title/Abstract] OR "non sebaceous lymphadenom*"[Title/Abstract] OR "Cancer of Salivary Gland"[Title/Abstract] OR "Cancer of the Salivary Gland"[Title/Abstract] OR "salivary gland cance*"[Title/Abstract] OR "sebaceous lymphadenom*"[Title/Abstract] OR "parotid neoplas*"[Title/Abstract] OR "Cancer of Parotid"[Title/Abstract] OR "Cancer of the Parotid"[Title/Abstract] OR "parotid cance*"[Title/Abstract] OR "sublingual gland neoplas*"[Title/Abstract] OR "submandibular gland neoplas*"[Title/Abstract] OR "tongue neoplas*"[Title/Abstract] OR "Cancer of Tongue"[Title/Abstract] OR "Cancer of the Tongue"[Title/Abstract] OR "tongue cance*"[Title/Abstract] OR "oral carcinom*"[Title/Abstract] OR "oral squamous cell carcinom*"[Title/Abstract] OR "buccal mucosa tum*"[Title/Abstract] OR "intraoral tum*"[Title/Abstract] OR "mouth tum*"[Title/Abstract] OR "oral cavity tum*"[Title/Abstract] OR "oral mucosa tum*"[Title/Abstract] OR "oral tum*"[Title/Abstract] | | |  |
| #7 | | "Nose Neoplasms"[MeSH Terms] OR "nose neoplas*"[Title/Abstract] OR "nasal neoplas*"[Title/Abstract] OR "Cancer of Nose"[Title/Abstract] OR "nose cance*"[Title/Abstract] OR "nasal cance*"[Title/Abstract] OR "Cancer of the Nose"[Title/Abstract] OR "paranasal sinus neoplas*"[Title/Abstract] OR "paranasal sinus cance*"[Title/Abstract] OR "cancer of paranasal sinus*"[Title/Abstract] OR "maxillary sinus neoplas*"[Title/Abstract] OR "cancer of maxillary sinus*"[Title/Abstract] OR "maxillary sinus cance*"[Title/Abstract] OR "sinonasal carcinom*"[Title/Abstract] OR "nasal and paranasal tum*"[Title/Abstract] OR "nasal tum*"[Title/Abstract] OR "nose tum*"[Title/Abstract] OR "sinonasal tum*"[Title/Abstract] OR "tumor of the nose"[Title/Abstract] | | |  |
| #8 | | #1 OR #2 OR #3 OR #4 OR #5 OR #6 OR #7 | | |  |
| #9 | | "Mesenchymal Stem Cells"[MeSH Terms] OR "mesenchymal stem cel*"[Title/Abstract] OR "bone marrow mesenchymal stem cel*"[Title/Abstract] OR "bone marrow stromal cel*"[Title/Abstract] OR "multipotent bone marrow stromal cel*"[Title/Abstract] OR "adipose derived mesenchymal stem cel*"[Title/Abstract] OR "adipose derived mesenchymal stem cel*"[Title/Abstract] OR "adipose derived mesenchymal stromal cel*"[Title/Abstract] OR "adipose derived mesenchymal stromal cel*"[Title/Abstract] OR "adipose tissue derived mesenchymal stem cel*"[Title/Abstract] OR "adipose tissue derived mesenchymal stem cel*"[Title/Abstract] OR "adipose tissue derived mesenchymal stromal cel*"[Title/Abstract] OR "adipose tissue derived mesenchymal stromal cel*"[Title/Abstract] OR "mesenchymal stromal cel*"[Title/Abstract] OR "multipotent mesenchymal stromal cel*"[Title/Abstract] OR "mesenchymal progenitor cel*"[Title/Abstract] OR "whartons jelly cel*"[Title/Abstract] OR "wharton s jelly cel*"[Title/Abstract] OR "bone marrow stromal stem cel*"[Title/Abstract] OR "MSC"[Title/Abstract] OR "MSCs"[Title/Abstract] | | |  |
| #10 | | #8 AND #9 | | |  |
|  | | | Embase | | |
| #1 | | | "Squamous Cell Carcinoma of Head and Neck":ti,ab,kw OR "head and neck squamous cell carcinom*":ti,ab,kw OR "Squamous Cell Carcinoma of the Head and Neck":ti,ab,kw OR "Squamous Cell Carcinoma of the Larynx":ti,ab,kw OR "Laryngeal Squamous Cell Carcinom*":ti,ab,kw OR "Squamous Cell Carcinoma of Larynx":ti,ab,kw OR "Squamous Cell Carcinoma of the Nasal Cavity":ti,ab,kw OR "Oral Tongue Squamous Cell Carcinom*":ti,ab,kw OR "Hypopharyngeal Squamous Cell Carcinom*":ti,ab,kw OR "Oral Cavity Squamous Cell Carcinom*":ti,ab,kw OR "Squamous Cell Carcinoma of the Mouth":ti,ab,kw OR "Oropharyngeal Squamous Cell Carcinom*":ti,ab,kw | | |
| #2 | | | "nasopharynx tumor"/exp OR "Nasopharyngeal Neoplas*":ti,ab,kw OR "nasopharynx neoplas*":ti,ab,kw OR "Cancer of Nasopharynx":ti,ab,kw OR "nasopharynx cance*":ti,ab,kw OR "nasopharyngeal cance*":ti,ab,kw OR "Cancer of the Nasopharynx":ti,ab,kw OR "nasopharyngeal carcinom*":ti,ab,kw OR "nasopharynx tum*":ti,ab,kw OR "nasopharyngeal tum*":ti,ab,kw OR "rhinopharyngeal tum*":ti,ab,kw OR "rhinopharynx tum*":ti,ab,kw OR "nasopharynx tum*":ti,ab,kw OR "neoplasm of the nasopharynx":ti,ab,kw OR "tumor of the nasopharynx":ti,ab,kw OR "tumor of the rhinopharynx":ti,ab,kw | | |
| #3 | | | "oropharynx tumor"/exp OR "oropharyngeal neoplas*":ti,ab,kw OR "oropharynx neoplas*":ti,ab,kw OR "oropharnyx cance*":ti,ab,kw OR "oropharyngeal cance*":ti,ab,kw OR "Cancer of the Oropharynx":ti,ab,kw OR "tonsil neoplas*":ti,ab,kw OR "tonsillar neoplas*":ti,ab,kw OR "Cancer of Tonsil":ti,ab,kw OR "Cancer of the Tonsil":ti,ab,kw OR "tonsil cance*":ti,ab,kw OR "tonsillar cance*":ti,ab,kw OR “tonsil carcinom*”:ti,ab,kw OR "oropharyngeal tum*":ti,ab,kw OR "oropharynx tum*":ti,ab,kw | | |
| #4 | | | "hypopharynx tumor"/exp OR "hypopharyngeal neoplas*":ti,ab,kw OR "hypopharyngeal cance*":ti,ab,kw OR "hypopharyngeal carcinom*":ti,ab,kw OR "hypopharynx cance*":ti,ab,kw OR "hypopharyngeal tum*":ti,ab,kw OR "hypopharynx tum*":ti,ab,kw | | |
| #5 | | | "larynx tumor"/exp OR "laryngeal neoplas*":ti,ab,kw OR "larynx neoplas*":ti,ab,kw OR "Cancer of Larynx":ti,ab,kw OR "larynx cance*":ti,ab,kw OR "laryngeal cance*":ti,ab,kw OR "Cancer of the Larynx":ti,ab,kw OR "throat cance*":ti,ab,kw OR "laryngeal carcinom*":ti,ab,kw OR "laryngocarcinom*":ti,ab,kw OR "glottic tum*":ti,ab,kw OR "laryngeal tum*":ti,ab,kw OR "larynx tum*":ti,ab,kw OR "neoplastic laryn*":ti,ab,kw OR "subglottic tum*":ti,ab,kw OR "supraglottic neoplas*":ti,ab,kw OR "vocal cord tum*":ti,ab,kw OR "vocal cord neoplas*":ti,ab,kw | | |
| #6 | | | "mouth tumor"/exp OR "mouth neoplas*":ti,ab,kw OR "oral neoplas*":ti,ab,kw OR "Cancer of Mouth":ti,ab,kw OR "oral cance*":ti,ab,kw OR "Cancer of the Mouth":ti,ab,kw OR "mouth cance*":ti,ab,kw OR "gingival neoplas*":ti,ab,kw OR "congenital epuli*":ti,ab,kw OR "oral leukoplaki*":ti,ab,kw OR "oral leukokeratos*":ti,ab,kw OR "oral keratos*":ti,ab,kw OR "hairy leukoplaki*":ti,ab,kw OR "oral hairy leukoplaki*":ti,ab,kw OR "lip neoplas*":ti,ab,kw OR "Cancer of Lip":ti,ab,kw OR "lip cance*":ti,ab,kw OR "palatal neoplas*":ti,ab,kw OR "salivary gland neoplas*":ti,ab,kw OR "salivary gland lymphadenom*":ti,ab,kw OR "non sebaceous lymphadenom*":ti,ab,kw OR "Cancer of Salivary Gland":ti,ab,kw OR "Cancer of the Salivary Gland":ti,ab,kw OR "salivary gland cance*":ti,ab,kw OR "sebaceous lymphadenom*":ti,ab,kw OR "parotid neoplas*":ti,ab,kw OR "Cancer of Parotid":ti,ab,kw OR "Cancer of the Parotid":ti,ab,kw OR "parotid cance*":ti,ab,kw OR "sublingual gland neoplas*":ti,ab,kw OR "submandibular gland neoplas*":ti,ab,kw OR "tongue neoplas*":ti,ab,kw OR "Cancer of Tongue":ti,ab,kw OR "Cancer of the Tongue":ti,ab,kw OR "tongue cance*":ti,ab,kw OR "oral carcinom*":ti,ab,kw OR "oral squamous cell carcinom*":ti,ab,kw OR "buccal mucosa tum*":ti,ab,kw OR "intraoral tum*":ti,ab,kw OR "mouth cavity tum*":ti,ab,kw OR "mouth tum*":ti,ab,kw OR "oral cavity tum*":ti,ab,kw OR "oral mucosa tum*":ti,ab,kw OR "oral tum*":ti,ab,kw | | |
| #7 | | | "nose tumor"/exp OR "nose neoplas*":ti,ab,kw OR "nasal neoplas*":ti,ab,kw OR "Cancer of Nose":ti,ab,kw OR "nose cance*":ti,ab,kw OR "nasal cance*":ti,ab,kw OR "Cancer of the Nose":ti,ab,kw OR "paranasal sinus neoplas*":ti,ab,kw OR "paranasal sinus cance*":ti,ab,kw OR "cancer of paranasal sinus*":ti,ab,kw OR "maxillary sinus neoplas*":ti,ab,kw OR "cancer of maxillary sinus*":ti,ab,kw OR "maxillary sinus cance*":ti,ab,kw OR "sinonasal carcinom*":ti,ab,kw OR "nasal and paranasal sinus tum*":ti,ab,kw OR "nasal and paranasal sinuses tum*":ti,ab,kw OR "nasal and paranasal tum*":ti,ab,kw OR "nasal cavity and paranasal sinus tum*":ti,ab,kw OR "nasal cavity and paranasal sinuses tum*":ti,ab,kw OR "nasal tum*":ti,ab,kw OR "nose tum*":ti,ab,kw OR "sinonasal tum*":ti,ab,kw OR "tumor of the nose":ti,ab,kw | | |
| #8 | | | #1 OR #2 OR #3 OR #4 OR #5 OR #6 OR #7 | | |
| #9 | | | "Mesenchymal Stem Cell"/exp OR "mesenchymal stem cel*":ti,ab,kw OR "bone marrow mesenchymal stem cel*":ti,ab,kw OR "bone marrow stromal cel*":ti,ab,kw OR "multipotent bone marrow stromal cel*":ti,ab,kw OR "adipose derived mesenchymal stem cel*":ti,ab,kw OR "adipose derived mesenchymal stem cel*":ti,ab,kw OR "adipose derived mesenchymal stromal cel*":ti,ab,kw OR "adipose derived mesenchymal stromal cel*":ti,ab,kw OR "adipose tissue derived mesenchymal stem cel*":ti,ab,kw OR "adipose tissue derived mesenchymal stem cel*":ti,ab,kw OR "adipose tissue derived mesenchymal stromal cel*":ti,ab,kw OR "adipose tissue derived mesenchymal stromal cel*":ti,ab,kw OR "mesenchymal stromal cel*":ti,ab,kw OR "multipotent mesenchymal stromal cel*":ti,ab,kw OR "mesenchymal progenitor cel*":ti,ab,kw OR "whartons jelly cel*":ti,ab,kw OR "wharton s jelly cel*":ti,ab,kw OR "bone marrow stromal stem cel*":ti,ab,kw OR "MSC":ti,ab,kw OR "MSCs":ti,ab,kw | | |
| #10 | | | #8 AND #9 | | |
|  | | Web of Science | |  |  |
| #1 | | TS=("Squamous Cell Carcinoma of Head and Neck" OR "head and neck squamous cell carcinom*" OR "Squamous Cell Carcinoma of the Head and Neck" OR "Squamous Cell Carcinoma of the Larynx" OR "Laryngeal Squamous Cell Carcinom*" OR "Squamous Cell Carcinoma of Larynx" OR "Squamous Cell Carcinoma of the Nasal Cavity" OR "Oral Tongue Squamous Cell Carcinom*" OR "Hypopharyngeal Squamous Cell Carcinom*" OR "Oral Cavity Squamous Cell Carcinom*" OR "Squamous Cell Carcinoma of the Mouth" OR "Oropharyngeal Squamous Cell Carcinom*") | |  |  |
| #2 | | TS=("nasopharynx tumor" OR "Nasopharyngeal Neoplas*" OR "nasopharynx neoplas*" OR "Cancer of Nasopharynx" OR "nasopharynx cance*" OR "nasopharyngeal cance*" OR "Cancer of the Nasopharynx" OR "nasopharyngeal carcinom*" OR "nasopharynx tum*" OR "nasopharyngeal tum*" OR "rhinopharyngeal tum*" OR "rhinopharynx tum*" OR "nasopharynx tum*" OR "neoplasm of the nasopharynx" OR "tumor of the nasopharynx" OR "tumor of the rhinopharynx") | |  |  |
| #3 | | TS=("oropharynx tumor" OR "oropharyngeal neoplas*" OR "oropharynx neoplas*" OR "oropharnyx cance*" OR "oropharyngeal cance*" OR "Cancer of the Oropharynx" OR "tonsil neoplas*" OR "tonsillar neoplas*" OR "Cancer of Tonsil" OR "Cancer of the Tonsil" OR "tonsil cance*" OR "tonsillar cance*" OR “tonsil carcinom*” OR "oropharyngeal tum*" OR "oropharynx tum*") | |  |  |
| #4 | | TS=("hypopharynx tumor" OR "hypopharyngeal neoplas*" OR "hypopharyngeal cance*" OR "hypopharyngeal carcinom*" OR "hypopharynx cance*" OR "hypopharyngeal tum*" OR "hypopharynx tum*") | |  |  |
| #5 | | TS=("larynx tumor" OR "laryngeal neoplas*" OR "larynx neoplas*" OR "Cancer of Larynx" OR "larynx cance*" OR "laryngeal cance*" OR "Cancer of the Larynx" OR "throat cance*" OR "laryngeal carcinom*" OR "laryngocarcinom*" OR "glottic tum*" OR "laryngeal tum*" OR "larynx tum*" OR "neoplastic laryn*" OR "subglottic tum*" OR "supraglottic neoplas*" OR "vocal cord tum*" OR "vocal cord neoplas*") | |  |  |
| #6 | | TS=("mouth tumor" OR "mouth neoplas*" OR "oral neoplas*" OR "Cancer of Mouth" OR "oral cance*" OR "Cancer of the Mouth" OR "mouth cance*" OR "gingival neoplas*" OR "congenital epuli*" OR "oral leukoplaki*" OR "oral leukokeratos*" OR "oral keratos*" OR "hairy leukoplaki*" OR "oral hairy leukoplaki*" OR "lip neoplas*" OR "Cancer of Lip" OR "lip cance*" OR "palatal neoplas*" OR "salivary gland neoplas*" OR "salivary gland lymphadenom*" OR "non sebaceous lymphadenom*" OR "Cancer of Salivary Gland" OR "Cancer of the Salivary Gland" OR "salivary gland cance*" OR "sebaceous lymphadenom*" OR "parotid neoplas*" OR "Cancer of Parotid" OR "Cancer of the Parotid" OR "parotid cance*" OR "sublingual gland neoplas*" OR "submandibular gland neoplas*" OR "tongue neoplas*" OR "Cancer of Tongue" OR "Cancer of the Tongue" OR "tongue cance*" OR "oral carcinom*" OR "oral squamous cell carcinom*" OR "buccal mucosa tum*" OR "intraoral tum*" OR "mouth cavity tum*" OR "mouth tum*" OR "oral cavity tum*" OR "oral mucosa tum*" OR "oral tum*") | |  |  |
| #7 | | TS=("nose tumor" OR "nose neoplas*" OR "nasal neoplas*" OR "Cancer of Nose" OR "nose cance*" OR "nasal cance*" OR "Cancer of the Nose" OR "paranasal sinus neoplas*" OR "paranasal sinus cance*" OR "cancer of paranasal sinus*" OR "maxillary sinus neoplas*" OR "cancer of maxillary sinus*" OR "maxillary sinus cance*" OR "sinonasal carcinom*" OR "nasal and paranasal sinus tum*" OR "nasal and paranasal sinuses tum*" OR "nasal and paranasal tum*" OR "nasal cavity and paranasal sinus tum*" OR "nasal cavity and paranasal sinuses tum*" OR "nasal tum*" OR "nose tum*" OR "sinonasal tum*" OR "tumor of the nose") | |  |  |
| #8 | | #1 OR #2 OR #3 OR #4 OR #5 OR #6 OR #7 | |  |  |
| #9 | | TS=("Mesenchymal Stem Cell" OR "mesenchymal stem cel*" OR "bone marrow mesenchymal stem cel*" OR "bone marrow stromal cel*" OR "multipotent bone marrow stromal cel*" OR "adipose derived mesenchymal stem cel*" OR "adipose derived mesenchymal stem cel*" OR "adipose derived mesenchymal stromal cel*" OR "adipose derived mesenchymal stromal cel*" OR "adipose tissue derived mesenchymal stem cel*" OR "adipose tissue derived mesenchymal stem cel*" OR "adipose tissue derived mesenchymal stromal cel*" OR "adipose tissue derived mesenchymal stromal cel*" OR "mesenchymal stromal cel*" OR "multipotent mesenchymal stromal cel*" OR "mesenchymal progenitor cel*" OR "whartons jelly cel*" OR "wharton s jelly cel*" OR "bone marrow stromal stem cel*" OR "MSC" OR "MSCs") | |  |  |
| #10 | | #8 AND #9 | |  |  |

## Supplementary Figure S1. Sensitivity analysis of the effect of MSCs on EMT markers in OSCC


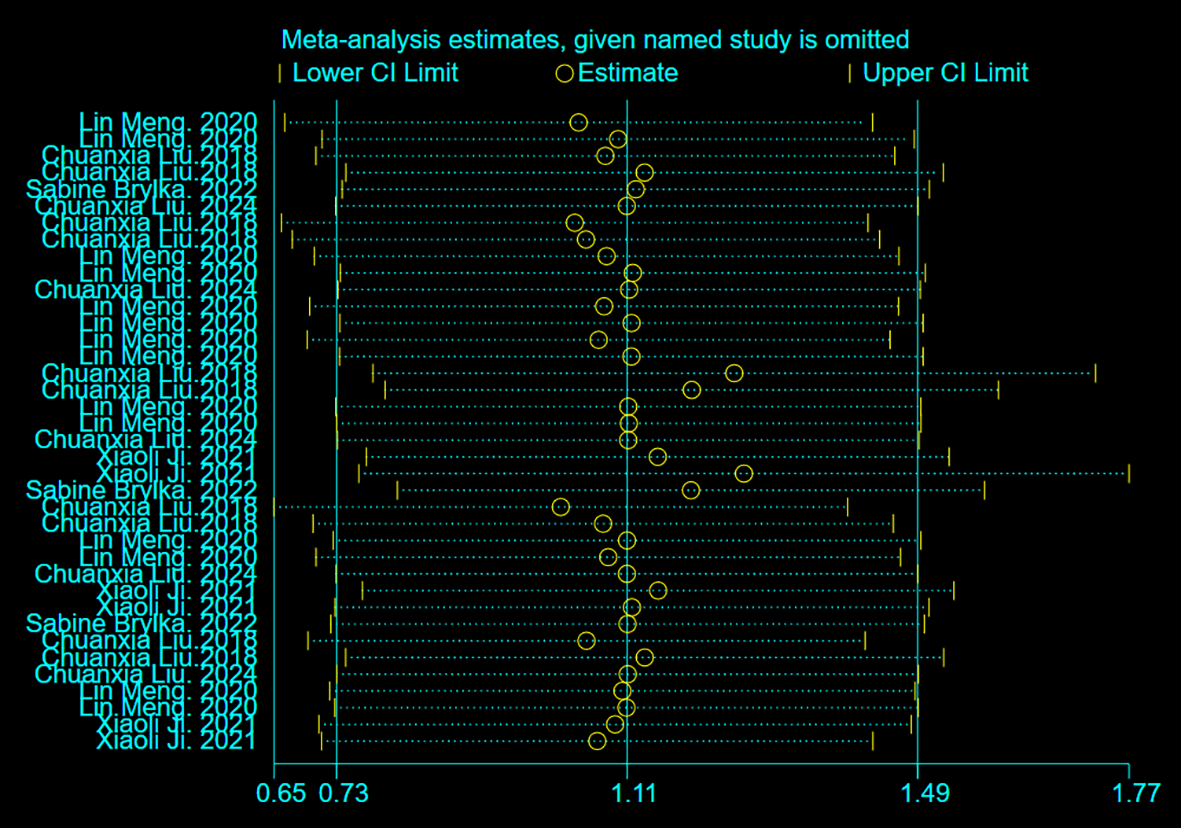


## Supplementary Figure S2. Sensitivity analysis of the effect of MSCs on EMT markers in NPC


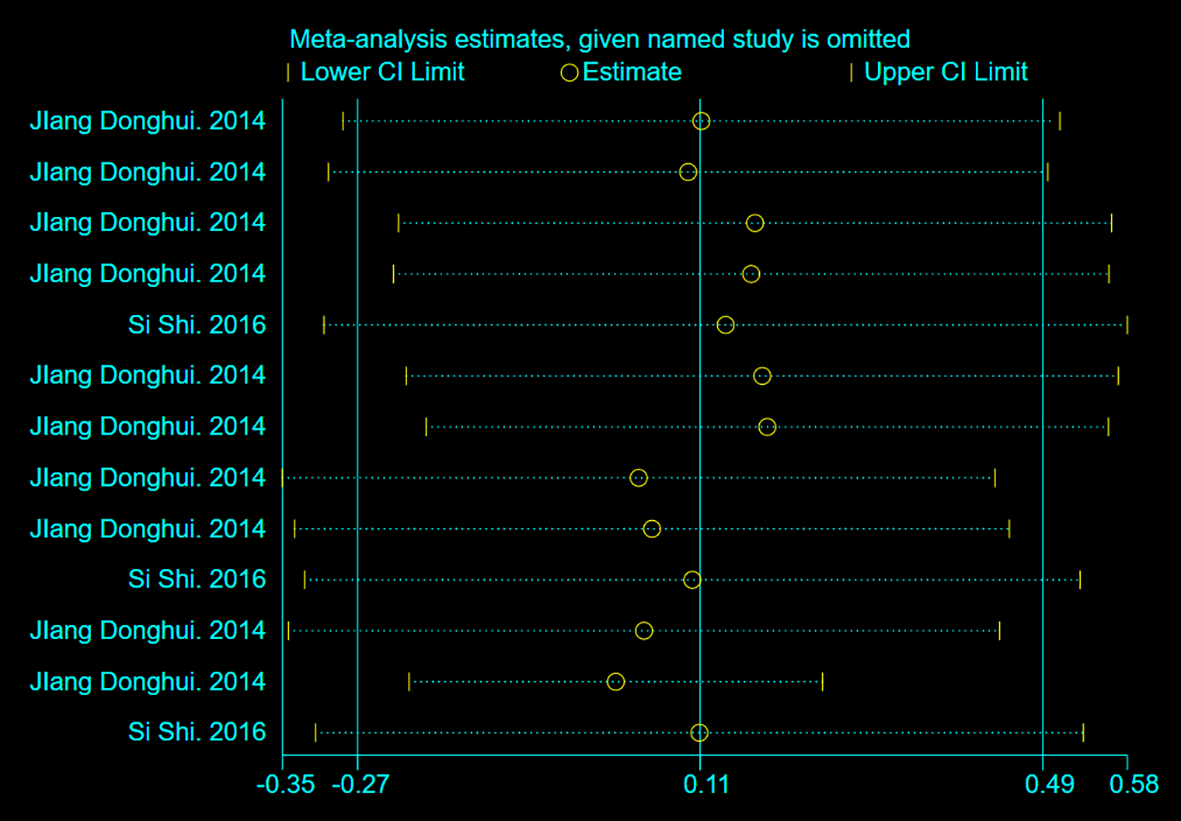


## Supplementary Table 2 Basic characteristics of inclusion research

| First Author, Year | Head and Neck Squamous Cell Carcinoma, HNSCC | Cell Line | Intervention | Cell source | Intervention Method | Biomarkers and related transcription factors of epithelial-mesenchymal transition | Changes in biomarkers | Biomarker detection methods |
| --- | --- | --- | --- | --- | --- | --- | --- | --- |
| Xiaoli Ji, 2021^[21]^ | oral squamous cell carcinoma, OSCC | CAL27 | mesenchymal stem cells, MSC | human oral squamous cell carcinoma | Indirect co-culture + conditioned medium | E-cadherin | ⬇ | Western blot |
|  |  |  |  |  |  | Vimentin | No differences |  |
|  |  |  |  |  |  | N-cadherin | ⬆ |  |
|  |  | WSU-HN6 |  |  |  | E-cadherin | ⬇ | Western blot |
|  |  |  |  |  |  | Vimentin | No differences |  |
|  |  |  |  |  |  | N-cadherin | ⬆ |  |
| JIang Donghui, 2014^[22]^ | Nasopharyngeal carcinoma, NPC | CNE1 | mesenchymal stem cells, MSC | human bone marrow | conditioned medium | E-cadherin | ⬇ | RT-qPCR |
|  |  |  |  |  |  | β-catenin | ⬇ |  |
|  |  |  |  |  |  | N-cadherin | ⬆ |  |
|  |  |  |  |  |  | Vimentin | ⬆ |  |
|  |  |  |  |  |  | Snail | ⬆ |  |
|  |  | CNE2 |  |  |  | E-cadherin | ⬇ | RT-qPCR |
|  |  |  |  |  |  | β-catenin | ⬇ |  |
|  |  |  |  |  |  | N-cadherin | ⬆ |  |
|  |  |  |  |  |  | Vimentin | ⬆ |  |
|  |  |  |  |  |  | Snail | ⬆ |  |
| Chuanxia Liu, 2018^[23]^ | oral squamous cell carcinoma, OSCC | CAL-27 | mesenchymal stem cells, MSC | human bone marrow | conditioned medium | E-cadherin | ⬇ | RT-qPCR |
|  |  |  |  |  |  | N-cadherin | ⬆ |  |
|  |  |  |  |  |  | Vimentin | ⬆ |  |
|  |  |  |  |  |  | Snail | ⬆ |  |
|  |  |  |  |  |  | Twist | ⬆ |  |
|  |  | HN4 |  |  |  | E-cadherin | ⬇ | RT-qPCR |
|  |  |  |  |  |  | N-cadherin | ⬆ |  |
|  |  |  |  |  |  | Vimentin | ⬆ |  |
|  |  |  |  |  |  | Snail | ⬆ |  |
|  |  |  |  |  |  | Twist | ⬆ |  |
| Chuanxia Liu, 2024^[24]^ | oral squamous cell carcinoma, OSCC | SCC-25 | mesenchymal stem cells, MSC | human bone marrow | conditioned medium | E-cadherin | ⬇ | RT-qPCR |
|  |  |  |  |  |  | N-cadherin | ⬆ |  |
|  |  |  |  |  |  | Vimentin | ⬆ |  |
|  |  |  |  |  |  | Snail | ⬆ |  |
|  |  |  |  |  |  | Twist | ⬆ |  |
| Sabine Brylka, 2022^[25]^ | oral squamous cell carcinoma, OSCC | SCC-040 | mesenchymal stem cells, MSC | human | Direct co-culture | E-cadherin | ⬇ | Immunohistochemistry |
|  |  |  |  |  |  | Vimentin | ⬆ |  |
|  |  |  |  |  |  | Snail | ⬆ |  |
| Lin Meng, 2020^[26]^ | oral squamous cell carcinoma, OSCC | CAL-27 | mesenchymal stem cells, MSC | human bone marrow | conditioned medium | E-cadherin | ⬇ | RT-qPCR |
|  |  |  |  |  |  | Vimentin | ⬆ |  |
|  |  |  |  |  |  | N-cadherin | ⬆ |  |
|  |  |  |  |  |  | Snail | ⬆ |  |
|  |  |  |  |  |  | ZEB1 | ⬆ |  |
|  |  |  |  |  |  | ZEB2 | ⬆ |  |
|  |  |  |  |  |  | Twist | ⬆ |  |
|  |  | FaDu |  |  |  | E-cadherin | ⬇ | RT-qPCR |
|  |  |  |  |  |  | Vimentin | ⬆ |  |
|  |  |  |  |  |  | N-cadherin | ⬆ |  |
|  |  |  |  |  |  | Snail | ⬆ |  |
|  |  |  |  |  |  | ZEB1 | ⬆ |  |
|  |  |  |  |  |  | ZEB2 | ⬆ |  |
|  |  |  |  |  |  | Twist | ⬆ |  |
| Si Shi, 2016^[17]^ | Nasopharyngeal carcinoma, NPC | CNE2 | mesenchymal stem cells, MSC | human bone marrow | Exosome | E-cadherin | ⬇ | Western blot |
|  |  |  |  |  |  | N-cadherin | ⬆ |  |
|  |  |  |  |  |  | Vimentin | ⬆ |  |
| Yu-Ling Wu, 2017^[27]^ | Tongue squamous cell carcinoma, TSCC | TSCCA | mesenchymal stem cells, MSC | human bone marrow | conditioned medium | E-cadherin | ⬇ | RT-qPCR |
|  |  |  |  |  |  | N-cadherin | ⬆ |  |
|  |  |  |  |  |  | Vimentin | ⬆ |  |
|  |  |  |  |  |  | Twist | ⬆ |  |
|  |  |  |  |  |  | MMP2 | ⬆ |  |
|  |  |  |  |  |  | MMP9 | ⬆ |  |
|  |  | CAL-27 |  |  |  | E-cadherin | ⬇ | RT-qPCR |
|  |  |  |  |  |  | N-cadherin | ⬆ |  |
|  |  |  |  |  |  | Vimentin | ⬆ |  |
|  |  |  |  |  |  | Twist | ⬆ |  |
|  |  |  |  |  |  | MMP2 | ⬆ |  |
|  |  |  |  |  |  | MMP9 | ⬆ |  |
